# Secondhand Smoking and Sudden Infant Death Syndrome: How can *in Silico* Pharmacokinetics and Circulation Models Contribute?

**DOI:** 10.3389/fbioe.2021.820404

**Published:** 2022-01-17

**Authors:** Harvey Ho, Tingting Ran, Xiaojuan Ji

**Affiliations:** ^1^ Auckland Bioengineering Institute, The University of Auckland, Auckland, New Zealand; ^2^ Children’s Hospital of Chongqing Medical University, Chongqing, China; ^3^ National Clinical Research Center for Child Health and Disorders, Chongqing Key Laboratory of Pediatrics, Chongqing, China

**Keywords:** SIDS, secondhand smoking, nicotine, pharmacokinetics, computer model

## Background

Sudden Infant Death Syndrome (SIDS) is the leading cause of death in infants in developed countries (Moon et al., 2007) (Rogers, 2009). The cardio-respiratory distress due to infant sleep position was considered as the leading risk factor that causes arousal and/or auto-resuscitation deficiency under hypoxia conditions (Moon et al., 2007). The “Back to sleep” campaign of the early 1990s recommended placing sleep babies in a supine position. The campaign led to a substantial decrease in SIDS cases (Rogers, 2009). However, nearly 3 decades later, SIDS remains one of the leading causes of infant death. Among many risk factors, exposure to passive or secondhand smoking (SHS) pre- and postpartum has attracted many research studies (Mitchell et al., 1997) (McMartin et al., 2002) (Albuquerque et al., 2004) (Raghuveer et al., 2016) (Tang et al., 2020). Indeed, SHS exposure and maternal smoking are considered the most modifiable risk factor associated with SIDS (Cnattingius, 2004). It is estimated that if all fetuses had not been exposed to maternal smoking *in utero,* about one-third of all SIDS deaths might have been prevented (Mitchell et al., 1997).

Blood flow measurements in fetuses and neonates seem to support the viewpoint. The systolic to diastolic (S/D) ratio in the fetal umbilical and middle cerebral arteries is greater in those exposed to maternal smoking than those without exposure (Albuquerque et al., 2004). High S/D ratios are associated with a lower or absent end-diastolic flow, which is typical in fetuses with intrauterine growth restriction (IUGR) (Albuquerque et al., 2004). More direct pieces of evidence are the measurements of nicotine and cotinine (the major metabolite of nicotine) in biopsy tissues of SIDS and non-SIDS victims (McMartin et al., 2002) (Milerad et al., 1994). From the 73 lung samples, McMartin and his co-workers found that there was a significantly higher nicotine concentration in the SIDS cases (19.64 ± 2.61 ng/g) than in non-SIDS cases (7.86 ± 1.63 ng/g). In contrast, there was no significant difference in cotinine concentrations between SIDS (10.87 ± 2.32 ng/g) and non-SIDS cases (8.71 ± 1.47 ng/g). In another study (Milerad et al., 1994), it was found that in the pericardial fluid of SIDS victims, about 70% of the 24 consecutively autopsied cases were moderately (10–20 ng) or heavily (20–50 ng) exposed to SHS.

Early intervention is critical for the prevention of SIDS. However, clinical investigations are hampered by the inaccessibility of fetal, neonatal, and infant tissues and *in vivo* measurements. Alternatively, animal models, e.g. mice models are used to investigate the effects of SHS (Hu et al., 2012) (Calton et al., 2016). It was found that SHS exposure induced a reduction in the ability of the pre-Bötzinger Complex, a region of inspiratory rhythm generation (Ramirez et al., 2012), to maintain a respiratory rhythm during exposure to severe hypoxia (Hu et al., 2012). However, it is still difficult to verify the underlying mechanisms in animal experiments that involve interweaving factors, as only a limited set of parameters could be tested.

Another approach, *in silico* modelling, is valuable because it can test the various hypothesis in a virtual setup. Still, few *in silico* models are developed for SIDS. A previous article proposed a roadmap for investigating the effects of maternal smoking on cardiovascular risks (Ho et al., 2020). In that roadmap, several components were suggested, including physiologically based pharmacokinetic (PBPK) models, blood flow and endothelial cell signaling models, bioinformatics, stem cell technology, etc. In this Opinion paper, we focus on two kinds of models, i.e. PBPK and circulation models, and their potential applications in SIDS research. We provide a light literature review of existing models, and discuss how they may be customised for the research of SIDS. We also discuss the importance of preparing fundamental data, e.g. longitudinal blood flow to key organs, that are still absent but crucial for developing these models.

## Paediatric Pharmacokinetic Models

The purpose of pharmacokinetic models is to quantify the absorption, distribution, metabolism, and excretion (ADME) of a drug/compound in the body. PBPK models use a group of compartments to represent blood, tissues (fat, muscle), and key organs. Differential equations are formulated to model the changes in the amount or concentration of a compound in that compartment (Jones and Rowland-Yeo, 2013). Paediatric PBPK (*p*-PBPK) models incorporate the physiological and metabolic differences between adults and children, such as the blood flow distribution in individual organs, age-dependent enzyme activities and renal clearance (Amice et al., 2021) (Zhang et al., 2020).

PBPK models of nicotine and cotinine have been recently reported for pregnant women (Amice et al., 2021). However, few *in silico* studies report the clearance of nicotine and cotinine, the major metabolite of nicotine, in neonates and infants (Gentry et al., 2003). Several factors need to be considered when extrapolating the current models of nicotine metabolism in adults to very young children and fetuses (Moon et al., 2007): neonates and infants have a limited CYP2A6 (the major enzyme that transforms nicotine into cotinine) activity (Jiang et al., 2013); (Rogers, 2009) the volume ratio of brain vs body in fetus/infants is much higher than adults (Mitchell et al., 1997); different circulation routes in fetuses, and the roles played by the placenta and amniotic fluid during pregnancy (Abduljalil et al., 2012). Nicotine can enter the plasma of neonates and infants via inhalation, i.e. through the lung, and also be via breastfeeding through the stomach and intestine (McMartin et al., 2002). P-PBPK models, therefore, need to be adjusted accordingly.

In brief, *in silico* models are valuable in assessing the absorption and clearance of nicotine in fetuses, neonates, and infants exposed to SHS and the risk of SIDS. Indeed, *in silico* models may sometimes be the only feasible way. For example, in the case of fetuses, the current clinical practice of sample taking is by analysing blood samples extracted from the umbilical cord but lacking any data from other organs (Huang et al., 2017). *In silico* models that predict nicotine concentration at early pregnancy or fetal organs like the fetal brain can have a unique value.

It is worth pointing out that parameters are critical in pharmacokinetic models. However, some parameters such as the volume of distribution and the hepatic and renal clearance are often unavailable. Therefore, computational methods, including linear or nonlinear regression, global parameter analysis such as the Latin Hypercube Sampling analysis, are used to fit model results to reported data (Zhang et al., 2020) (Jiang et al., 2013).

## Circulation Models

Circulation models are used to investigate the hemodynamics and regulation mechanisms of the cardiovascular system under both healthy and diseased states (Gao et al., 1998). Many computational methods and techniques have been proposed for circulation modelling. They may be roughly grouped according to the spatial dimensions (from 0D to 3D) of the governing equations. For example, 0D models use electronic components such as resistors to represent major arteries and veins, where the temporal blood pressure and flow rate are simulated (Gao et al., 1998). In contrast, 1D to 3D models take spatial dimensions of blood vessels (longitudinal and radial dimensions) into the equation, in addition to the time variable (Ho et al., 2009). Models of different dimensions can be combined. For example, a multiscale 0D-3D model was developed where 0D models were used to represent the systemic, coronary, and pulmonary circulations, whereas the 3D model was used for the systemic-to-pulmonary shunt (Migliavacca et al., 2006).

The regulation of the cardiovascular system from SIDS is still not well understood. The most relevant vessels to SIDS may be those in the heart, the brain, and the lung. Although many cerebral circulation models have been developed for adults, e.g. in (Gao et al., 1998) (Ho et al., 2009), few cerebral circulation models have been developed for infants. The infant’s brain has a similar vascular morphology to adults, though vessel dimensions and electronic component parameters need to be rescaled. In the case of SIDS, the blood supply to the brain stem via the basilar artery can be critical since the regulation centre of respiratory rate exists in the pons, and a gasping centre is thought to be located in the ventrolateral medulla (St.-John et al., 2009).

*In silico* circulation models could help by testing the hypothesis of poor blood supply, thus hypoxia, associated with SIDS. For example, increased cerebral blood flow velocities were reported in neonates of smoking mothers (Abdul-Khaliq et al., 1993), which can be used as a clue for circulation models. A relevant example is the fetal circulation model of (Garcia-Canadilla et al., 2014) that investigates the blood supply to the brains of IUGR subjects, where flow is redistributed to maintain cerebral supply (brain sparing) of different degrees of severity. The hypothesis of poor blood supply to the basilar artery can be simulated by the high resistance of its afferent vessels, the vertebral arteries. This in turn affects the distribution of the cerebral circulation in neonates and infants. Verification of the hypothesis depends upon flow data, which are usually collected from Doppler ultrasonography.

## Blood Flow Data

The blood flows in fetuses exposed to maternal smoking were measured by the pulsed Doppler method for large cerebral vessels, as well as umbilical vessels (Albuquerque et al., 2004). Cerebral blood flow velocities were also measured for neonates of smoking mothers (Abdul-Khaliq et al., 1993). In the study of (Abdul-Khaliq et al., 1993), it was found that the systolic, mean, and diastolic flow velocity in the anterior, internal carotid, and basilar cerebral arteries of neonates (20–42 h old) were significantly higher than those in the control group, despite their lower birth weight, gestational age, and systolic blood pressure.

However, flow velocities (in cm/s) in blood vessels cannot be translated into flow rates (in mL/s), which is calculated by the product of the flow velocity and the cross-section area. The data for the latter requires measuring the diameter of the artery under the B-mode of ultrasonic imaging. Therefore, both flow velocities and blood vessel diameters are required to verify that the blood supply to the brain stem is an important trigger of SIDS. Unfortunately, literature reporting both flow velocities and vessel diameters in neonates and infant are scant. Even more scarce are the developmental, longitudinal data of the blood flow along with several key time points of infancy. These data are not only important for circulation models, but are also useful for PBPK models because blood flow rates are a key parameter in calculating the distribution of drugs/compounds into different organs.

## Discussion

In this small paper, we have discussed the potential use of *in silico* circulation and pharmacokinetics models in SIDS research, the modifications that need to be made, and the important data (such as longitudinal blood flow) that are still absent. While new evidence has been found regarding the connection between SHS and SIDS, *in silico* studies are far lagging behind. For example, abnormal pontine Kölliker–Fuse nucleus (KFN) developments are suspected to be related to SIDS (Lavezzi, 2015). Interestingly, the majority of mothers of infants with altered KFN development were found to be smokers (Lavezzi, 2015). The prevalence of smoking among women exceeds 10% in many developed countries (Dessì et al., 2018). In the USA, the prevalence of maternal smoking is about 14% (Dessì et al., 2018). A recent survey found that approximately 30% of pregnant women were exposed to SHS (more than 15 min per day) in a metropolitan city in China (Tang et al., 2020). In New Zealand, the indigenous Māori infants are six times more likely to die of SIDS than non-Māori New Zealanders (Blakely et al., 2004). Incidentally, Māori women have a much higher pregnancy smoking rate (32%) than mothers in other ethnic groups.

This public health concern calls for novel research methodology, including computational models. For cardiovascular models to assess relevant risk factors, important questions need to be asked, such as the cause of fetal hypoxia that impairs brain functions (Pastrakuljic et al., 1998) (Elhaik, 2016), the abnormal respiratory rhythm (Hu et al., 2012), the chronic/acute effects of nicotine on the cardiovascular system, etc. Before constructing a computer model, thoughts must be put into the physiological/pathological context. For SIDS, *in silico* pharmacokinetic and circulation models can only provide simulations from their perspectives that need to be placed into the large picture of SIDS. And, while modelling techniques are well established in these disciplines, questions such as which modelling methods are to be chosen, their validation methods are still very challenging. A take-for-granted model may misinterpret the underlying phenomena. In addition, while this paper discusses pharmacokinetic and circulation models only, there are certainly other *in silico* models, e.g. celluar or molecular kinetics models for hypoxic response could provide fresh ideas underneath SIDS. Numerical methods such as machine learning could assist data analysis such as patient-record of SIDS. Therefore, we believe a closer tie among clinicians, experimentalists and modellers, and a publicly available database of data (Table 1) and *in silico* models would greatly assist the research of SIDS.

**TABLE 1:** Clinical, experimental or epidemiologic data associated with SHS and SIDS mentioned in the paper.

Literature	Data	Reference
Milerad et al. (1994)	the pericardial fluid of SIDS victims	Milerad et al. (1994)
McMartin et al. (2002)	Lung tissue nicotine/cotinine concentration in SIDS and non-SIDS infants	McMartin et al. (2002)
Albuquerque et al. (2004)	Arterial velocity waveform of foetuses exposed to SHS	Albuquerque et al. (2004)
Blakely et al. (2004)	Ethnic mortality in infants	Blakely et al. (2004)
Hu et al. (2012)	Prenatal nicotine exposure of *in vitro* respiratory rhythm to hypoxia in mice	Hu et al. (2012)
Ramirez et al. (2012)	Activity of the pre-Bötzinger Complex of mice exposed to SHS	Ramirez et al. (2012)
Lavezzi (2015)	Pontine Kölliker-Fuse nucleus developments	Lavezzi, (2015)
Tang et al. (2020)	SHS survey among pregnant women in a metropolitan city in China	Tang et al. (2020)
